# Bioavailability of intravenous fosphenytoin sodium in healthy Japanese volunteers

**DOI:** 10.1007/s13318-012-0105-x

**Published:** 2012-09-12

**Authors:** Yushi Inoue, Naotaka Usui, Tadayuki Hiroki, Kenji Shimizu, Susumu Kobayashi, Shigeki Shimasaki

**Affiliations:** 1 National Epilepsy Center, Shizuoka Institute of Epilepsy and Neurological Disorders, Shizuoka, Japan; 2 Kurume Clinical Pharmacology Clinic, Fukuoka, Japan; 3 Nobelpharma Co., Ltd., Tokyo, Japan

**Keywords:** Fosphenytoin sodium injection, Phenytoin, Pharmacokinetics, Safety, Tolerability

## Abstract

To compare and evaluate the bioavailability for intravenous fosphenytoin sodium with that of intravenous phenytoin sodium in Japanese subjects. In study 1, healthy Japanese male volunteers received a 30-min infusion of 375 mg fosphenytoin sodium or an equimolar dose of 250 mg phenytoin by a double-blind, crossover method. In study 2, other healthy Japanese male volunteers received a 30-min or 10-min infusion of 563 mg fosphenytoin sodium, followed by a dose of 750 mg after 2 weeks in an unblinded manner. Comparing with 250 mg phenytoin sodium, 375 mg fosphenytoin sodium exhibited lower total plasma phenytoin *C*_max_, whereas the geometric mean ratio of the AUC of total and free phenyotoin for fosphenytoin sodium at a dose of 375 mg was very similar to phenytoin sodium at a equimolar dose of 250 mg (AUC_0–*t*_ ratio: 0.98 and 1.02, respectively). Therefore, fosphenytoin is almost completely converted to phenytoin in subjects. Fosphenytoin sodium was rapidly converted to phenytoin at doses of 375, 563, and 750 mg. The maximum concentration (*C*_max_) of total plasma phenytoin increased in a dose-dependent manner. The area under the plasma concentration–time curve (AUC) increased slightly more than proportionally with the administered dose, and clearance (CL) decreased with increasing dose. Pain and other infusion-site reactions were reported by all 12 subjects with phenytoin sodium, whereas very few symptoms were observed with fosphenytoin sodium. In conclusion, fosphenytoin sodium is considered to be a useful substitute for phenytoin sodium with almost no associated injection-site reactions.

## Introduction

Phenytoin sodium injection is used to treat convulsions especially in emergencies. It has been on the market for approximately 50 years in Japan and occupies an important position as a next-best therapeutic agent in the treatment of status epilepticus not responding to diazepam (Sugai 2007). This formulation is a hypertonic solution of an osmotic pressure ratio of approximately 29 to physiological saline with pH 12. Hence, it can cause tissue damage resulting in pain, phlebitis, and purple glove syndrome, and its use is associated with significant risks (Earnest et al. 1983; Hayes and Chesney 1993; Hanna 1992).

3-Phosphoryloxymethyl phenytoin disodium (fosphenytoin sodium) is a water-soluble phenytoin prodrug with an injectable preparation formulated at a pH of 8.5–9.1 and an osmotic pressure ratio of approximately 1.9. Fosphenytoin is rapidly and completely converted to phenytoin by phosphatases present in the liver, red blood cells, and many other tissues (Browne et al. 1996). The conversion half-life is 8–15 min; however, the exact site(s) of conversion has not been determined. The conversion occurs independent of prior phenytoin plasma concentration (Browne et al. 1993). Also, no factors have been found that may affect conversion, including race, gender or age.

The use of fosphenytoin sodium avoids problems associated with phenytoin sodium. The preparation is widely used in the USA, EU, and other countries. However, fosphenytoin sodium injections have not been developed/employed in Japan, and Japanese patients have therefore been unable to benefit from its use.

In the present study, fosphenytoin sodium was administered to healthy Japanese male volunteers with the primary aim of establishing pharmacokinetics, and secondarily to confirm safety and tolerability in support of the drug’s development in Japan.

Regarding pharmacokinetics, both total and free phenytoin concentrations were measured so that the influence of non-metabolized fosphenytoin on phenytoin protein binding could be investigated.

## Objective

The aim of the present study was to reveal the pharmacological profile of fosphenytoin sodium in comparison with phenytoin sodium in healthy Japanese volunteers.

## Methods

Studies 1 and 2 were conducted in the Kurume Clinical Pharmacology Clinic (Fukuoka, Japan) in accordance with International Conference on Harmonization of Good Clinical Practice after approval by the Institutional Review Board of the clinic. Informed consent was obtained from each subject before study initiation.

### Study 1

A randomized, double-blind, crossover study of 375 mg fosphenytoin sodium and 250 mg phenytoin sodium was conducted. Doses of test drugs were selected based on the following: (1) in Japan, the maximum allowable single dose of phenytoin sodium is 250 mg (as indicated on the package label), (2) fosphenytoin at a dose of 375 mg is equimolar to phenytoin sodium at 250 mg, (3) the same dosage design was employed in Browne’s study (Browne et al. 1989), so an indirect comparison of pharmacokinetic results between Japanese and Caucasian subjects could be made. Twelve subjects meeting the criteria outlined in Table 1 were divided into two groups (i.e., sequence 1 and 2) of six subjects each. In sequence 1 subjects, fosphenytoin sodium (375 mg, i.v.) was administered over 30 min in period 1, and phenytoin sodium (250 mg, i.v.) was administered over 30 min in period 2. In sequence 2 subjects, phenytoin sodium (250 mg, i.v.) was administered over 30 min in period 1, and fosphenytoin sodium (375 mg, i.v.) was administered over 30 min in period 2. A 2-week washout period was set between periods 1 and 2. Fosphenytoin sodium at a dose of 375 mg and phenytoin sodium at a dose of 250 mg are nearly equimolar, at 0.92 and 0.91 mmol, respectively. The infusion rate was 12.5 mg/min for fosphenytoin sodium and 8.3 mg/min for 250 mg phenytoin sodium.

**Table 1:** Inclusion and exclusion criteria for studies 1 and 2

*Inclusion criteria*	
1.	Healthy Japanese males aged 20–40 years
2.	Body weight: 55–80 kg, body mass index (BMI)^a^: 18.0–26.0 kg/m^2^
3.	Subjects judged to be eligible by a physician at the screening test carried out within 4 weeks before initial administration of the study drug
4.	Voluntarily willing to participate in this study and able to provide a signed informed consent form
*Exclusion criteria*	
1.	Hypersensitivity to any component of the study drug or hydantoin compounds
2.	Supine or standing blood pressure outside the range 100–140/60–90 mmHg at rest
3.	Resting heart rate <50 beats/min in supine and standing positions
4.	Apparently abnormal 12-lead ECG or 24-h Holter ECG
5.	Apparently abnormal vasovagal reflex during blood collection
6.	Hypoalbuminemia
7.	Apparent heart, lung, liver, kidney, blood, metabolic, digestive, brain, and psychoneurological (including convulsion) diseases; other diseases requiring treatment; abnormal thyroid function; and abnormal fasting blood glucose
8.	Hypersensitivity to any drugs or foods
9.	Previous intake of a meal containing Saint John’s wort within 7 days before each treatment period
10.	Previous intake of a meal containing grapefruit juice within 7 days before each treatment period
11.	Use of any medication (including OTC medication) within the last 7 days
12.	Participation in other clinical studies within the last 3 months
13.	Blood donation (blood collection) totaling ≥400 mL within the last 3 months
14.	Smoking history within the last 6 months
15.	Alcohol abuse or positive for drugs of abuse
16.	Positive results in immunoserological tests (e.g., HIV)
17.	Any other subjects judged by a physician to be ineligible for participation in this study

^a^ *BMI* weight (kg)/height^2^ (m^2^)

### Study 2

Twelve new subjects meeting the criteria outlined in Table 1 received a single intravenous dose of 563 mg fosphenytoin sodium (30-min infusion to 6 subjects and 10-min infusion to 6 subjects) randomly in unblinded fashion, followed by a single intravenous dose of 750 mg fosphenytoin sodium (30-min infusion to 6 subjects and 10-min infusion to 5 subjects) after a 2-week washout period. The infusion rate of 563 mg fosphenytoin sodium was 18.8 mg/min during the 30-min infusion and 56.3 mg/min during the 10-min infusion. The infusion rate of 750 mg fosphenytoin sodium was 25 mg/min during the 30-min infusion and 75 mg/min during the 10-min infusion. The dose increase of fosphenytoin sodium to 750 mg was made after carefully confirming safety at the lower dose. The maximum dose (750 mg) in study 2 was expected to become the minimum dose given to patients, particularly as a seizure prophylaxis.

In studies 1 and 2, total and free plasma phenytoin concentrations as well as total plasma fosphenytoin concentrations were determined for all subjects before administration, and at 10, 20, 30, 40, 50 min, and 1, 1.25, 1.5, 2, 4, 8, 12, 24, 48, and 72 h after the start of infusion.

Subjects were asked to assess infusion-site symptoms (pain, burning, itching) before administration, and then at 30 min, 1, 2, 4, 8, 12, 24, 48, and 72 h after the start of infusion, by rating their intensity on a 10-cm visual analog scale (VAS; 0 = none to 10 = most severe). Physicians assessed findings (swelling, erythema, and tenderness) observed in the region surrounding the infusion site by grading their intensity on a scale of “0 = none” to “10 = severe.” Furthermore, pulse oximeter oxygen saturation (SpO_2_), neurological symptoms, electrocardiography (ECG), and vital signs were monitored intensively from the start of infusion to 4 h after infusion and at regular intervals afterward.

Undesirable symptoms or abnormal findings observed after treatment administration were regarded as adverse events.

### Validation for assay

Samples for determination of drug concentration were frozen to −20 °C immediately after collection at the study site and transported to the Analysis Center (Sumika Chemical Analysis Service, Ltd., Osaka, Japan). In the Analysis Center, drug concentrations were determined by liquid chromatography coupled with tandem mass spectrometry. Interassay precision and accuracy were found to be excellent (Table 2).

**Table 2:** Performance characteristics for LC/MS/MS assay

	Total fosphenytoin	Total phenytoin	Free phenytoin
Internal standard	Dexamethasone sodium phosphate	Mephenytoin	Mephenytoin
Linearity (μg/mL)	0.4–200 μg/mL (*r* ≤ 0.9983)	0.1–50 μg/mL (*r* ≤ 0.9976)	0.04–20 μg/mL (*r* ≤ 0.9988)
Precision of interassay (%RSD)	4.6–5.2	3.6–6.4	5.2–10.0
Accuracy of interassay (% relative error)	−6.6 to −2.3	−10.1 to −3.4	−4.5 to −2.1

### Statistical analyses

#### Pharmacokinetic parameters

The *C*_max_ was determined by measuring maximum observed plasma concentrations from data without interpolation. The *t*_max_ was recorded as the time at which *C*_max_ occurred. The area under the plasma concentration–time curve from time zero to time *t* (AUC_0–*t*_), where *t* is the last time point with a measurable concentration for an individual formulation, was calculated by the trapezoidal method. The area under the plasma concentration–time curve from time zero to time infinity (AUC_0–∞_) was estimated using the equation:

1 $$ {\text{AUC}}_{0-\infty } = {\text{ AUC}}_{ 0-t} + \, C_{t} /\lambda {\text{z}} $$

where *C*_*t*_ is the last measurable drug concentration and λz is the terminal rate constant. Half-life (*t*_1/2_) was calculated as ln(2)/λz. Clearance (CL) was calculated as dose/AUC_0–∞_, and distribution volume (Vd) was calculated as CL/λz.

Dose proportionality of C_max_ and AUC was assessed by linear regression analysis as *Y* = α + β*X*, where *Y* represents the parameters and *X* is dose/kg weight. The coefficient of correlation (*r*) was also calculated using Pearson’s product-moment.

#### Comparisons of *C*_max_ and AUC_0–*t*_ between 375 mg fosphenytoin sodium and 250 mg phenytoin sodium for study 1

Analysis of variance was done on the common logarithms of AUC_0–*t*_ and *C*_max_ by the linear mixed effects model using the subject as a random effect, and sequence, period, and treatment as fixed effects. When the 90 % confidence interval (CI) of the ratio (fosphenytoin/phenytoin) of geometric mean was within 0.8–1.25 (US-FDA 2003; European Medicines Agency 2008), the two treatments were considered to be bioequivalent. The mean is expressed as mean ± standard deviation unless otherwise specified.

#### Profile of % unbound phenytoin

Analysis was done on the profile of % unbound phenytoin using the linear mixed effects model with subject as a random effect, and treatment, time, rate of infusion, treatment × time, rate × treatment and rate × time as fixed effects.

## Results

In a total of 12 subjects entered in study 1, 6 received 375 mg fosphenytoin sodium (sequence 1), while the other 6 received 250 mg phenytoin sodium (sequence 2) over a 30-min infusion in period 1. One subject who received phenytoin sodium dropped-out during the infusion procedure because of pain. Another subject who also received phenytoin sodium finished period 1, but showed moderate decreased blood pressure during the infusion procedure and was prevented by the investigator from proceeding to period 2. All 6 subjects in sequence 1 then advanced to period 2, where they received 250 mg phenytoin sodium over a 30-min infusion. Four subjects in sequence 2 were able to advance to period 2, where they received 375 mg fosphenytoin sodium over a 30-min infusion. In period 2, one subject who received phenytoin sodium dropped-out during the infusion procedure due to pain. Consequently, 9 out of 12 subjects completed both periods 1 and 2. One subject (sequence 1) finished only period 1 (administered fosphenytoin sodium), one subject (sequence 2) finished only period 1 (administered phenytoin sodium) and one subject (sequence 2) was not used in the pharmacokinetics analysis.

Twelve subjects other than those in study 1 received 563 mg fosphenytoin sodium (6 over a 10-min and 6 over a 30-min infusion) in the first step of study 2. However, 2 of the 6 subjects in the 10-min infusion group showed moderate adverse events and did not continue to the next step of 750 mg fosphenytoin sodium (10-min infusion group). Therefore, only one new subject was able to be enrolled to replace the subjects that dropped-out. Consequently, 11 subjects received 750 mg fosphenytoin sodium (5 in the 10-min and 6 in the 30-min infusion groups) in the late step of study 2.

No marked differences were observed in age, body height, body weight, and body mass index between subjects across study 1 and study 2 (Table 3).

**Table 3:** Demographic data

	Study 1		Study 2			
	PHT 250 mg	FOS 375 mg	FOS 563 mg		FOS 750 mg	
Infusion rate (mg/min) (duration of infusion)	8.3 (30 min)	12.5 (30 min)	18.75 (30 min)	56.3 (10 min)	25 (30 min)	75 (10 min)
No. of subjects	12	10^a^	6	6	6	5
*Age*						
Mean (years)	22.7	22.1	28	24.2	28	27.2
SD	2.7	1.4	6.3	5.6	6.3	6.5
Min–max	20–30	20–25	23–37	20–34	23–37	20–34
*Height*						
Mean (cm)	170	169	176	170	176	173
SD	5	4	5.7	5	5.7	4.6
Min–max	162–181	162–176	170–186	164–178	170–186	167–178
*Weight*						
Mean (kg)	64.6	64.5	65.3	64.3	65.3	63.7
SD	5.77	6.2	4.91	4.69	4.91	4.25
Min–max	56.9–74.4	56.9–74.4	58.1–71.7	58.8–70.0	58.1–71.7	58.8–69.8
*BMI*						
Mean (%)	22.25	22.5	21.03	22.15	21.03	21.28
SD	1.8	1.9	1.23	1.79	1.23	1.46
Min–max	19.6–25.7	19.6–25.7	19.8–22.9	19.2–24.2	19.8–22.9	19.2–23.2

*FOS* intravenous fosphenytoin sodium, *PHT* intravenous phenytoin sodium

^a^Two subjects were not returned to crossover treatment (period 2: fosphenytoin sodium treatment) because of adverse events

**Fig. 1:**
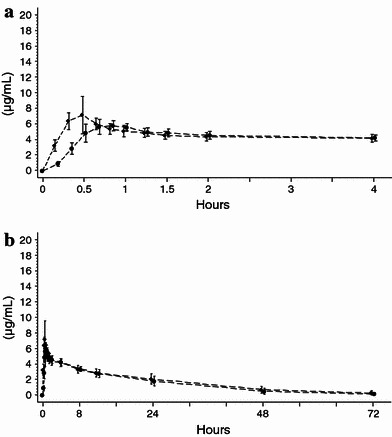
Mean (standard deviation) plasma total phenytoin concentration–time profiles for the first 4 h (**a**) and for 72 h (**b**) after intravenous administration of fosphenytoin sodium and phenytoin sodium in healthy Japanese subjects. *Closed circles* indicate fosphenytoin sodium 375 mg over a 30-min infusion (rate: 12.5 mg/min) for study 1 (*n* = 10). *Closed diamond* indicates phenytoin sodium 250 mg over a 30-min infusion (rate: 8.3 mg/min) for study 1 (*n* = 10)

The time courses for plasma total phenytoin concentration after intravenous administration of fosphenytoin sodium at a dose of 375 mg and phenytoin sodium at a dose of 250 mg are illustrated in Fig. 1. Very similar profiles of total phenytoin concentrations are shown for both fosphenytoin sodium and phenytoin sodium around 40 min after administration.

The *C*_max_ and AUC values for total and free phenytoin were compared in 9 subjects who completed both periods in study 1. The *C*_max_ values for total and free phenytoin were lower in subjects administered 375 mg fosphenytoin sodium than those administered 250 mg phenytoin sodium (Table 4). The AUC_0–*t*_ value was nearly identical in both treatment groups, and the geometric mean ratio and 90 % CI were 0.98 and 0.92–1.05 for total phenytoin and 1.02 and 0.89–1.16 for free phenytoin, respectively. The AUC_0–∞_ values for total and free phenytoin were also similar in both treatment groups (Table 4).

**Table 4:** Comparison of *C*_max_ and AUC between intravenous fosphenytoin sodium and intravenous phenytoin sodium in study 1

Parameter	Variable	Estimate	90 % Confidence interval	
			Lower	Upper
*Plasma total phenytoin*				
*C*_max_ (μg/mL)	FOS	5.83	5.21	6.51
	PHT	6.98	6.25	7.81
	Ratio	0.83	0.74	0.94
AUC_0–*t*_ (μg × h/mL)	FOS	100	89	112
	PHT	102	91	114
	Ratio	0.98	0.92	1.05*
AUC_0–∞_ (μg × h/mL)	FOS	110	*95*	*128*
	PHT	116	*99*	*134*
	Ratio	0.95	0.89	1.02*
*Plasma free phenytoin*				
*C*_max_ (μg/mL)	FOS	0.45	0.39	0.52
	PHT	0.51	0.44	0.58
	Ratio	0.89	0.74	1.06
AUC_0–*t*_ (μg × h/mL)	FOS	5.14	4.65	5.67
	PHT	5.05	4.57	5.57
	Ratio	1.02	0.89	1.16*
AUC_0–∞_ (μg × h/mL)	FOS	8.06	6.92	9.39
	PHT	7.95	6.83	9.27
	Ratio	1.01	0.84	1.22*

Estimates were calculated for nine subjects with paired data of FOS and PHT by the linear mixed effect model using subject as a random effect, and sequence, period, and treatment as fixed effects. Log-transformation of exposure measures were made for statistical analyses. Ratio: FOS/PHT

*Criteria for bioequivalence: 90 % confidence interval should be within the range 0.8–1.25

*FOS* intravenous fosphenytoin sodium 375 mg, *PHT* intravenous phenytoin sodium 250 mg

**Fig. 2:**
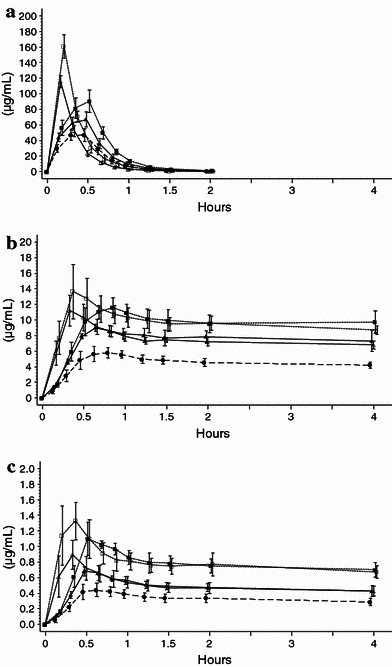
Mean (standard deviation) plasma total fosphenytoin (**a**), total phenytoin (**b**) and free phenytoin (**c**) concentration–time profiles for the first 4 h after intravenous administration of fosphenytoin sodium in healthy Japanese subjects. *Closed circles* indicates fosphenytoin sodium 375 mg over a 30-min infusion (rate: 12.5 mg/min) for study 1 (*n* = 10). *Closed triangles* indicates fosphenytoin sodium 563 mg over a 30-min infusion (rate: 18.8 mg/min) for study 2 (*n* = 6). *Open triangles* indicates fosphenytoin sodium 563 mg over a 10-min infusion (rate: 56.3 mg/min) for study 2 (*n* = 6). *Closed squares* indicate fosphenytoin sodium 750 mg over a 30-min infusion (rate: 25.0 mg/min) for study 2 (*n* = 6). *Open squares* indicates fosphenytoin sodium 750 mg over a 10-min infusion (rate: 75.0 mg/min) for study 2 (*n* = 5)

The time course and pharmacokinetic parameters for plasma total fosphenytoin and phenytoin concentrations, as well as free phenytoin concentrations by infusion rate after intravenous administration of fosphenytoin sodium at doses of 375, 563, and 750 mg are illustrated in Fig. 2 and Table 5.

**Table 5:** Pharmacokinetic parameters

	Drug: dose (IV rate)	*n*	*C*_max_ (μg/mL)	*T*_max_ (h)	*t*_1/2_(h)	AUC_0–*t*_ (μg × h/mL)	AUC_0–∞_ (μg × h/mL)	CL (L/h)	Vd (L)
Plasma total fosphenytoin	30-min infusion								
	FOS: 375 mg (12.5 mg/min)	10	49.4 (6.4)	0.40 (0.09)	0.24 (0.06)	30.8 (5.6)	31.0 (5.6)	11.1 (1.8)	3.76 (0.68)
	FOS: 563 mg (18.75 mg/min)	6	67.2 (9.4)	0.47 (0.07)	0.26 (0.05)	42.3 (6.8)	42.6 (6.9)	12.1 (2.1)	4.50 (0.56)
	FOS: 750 mg (25 mg/min)	6	90.4 (14.6)	0.53 (0.07)	0.28 (0.04)	56.1 (6.3)	56.5 (6.5)	12.0 (1.4)	4.85 (0.45)
	10-min duration of infusion								
	FOS: 563 mg (56.3 mg/min)	6	115.7 (7.6)	0.17 (0.00)	0.31 (0.07)	36.0 (2.6)	36.3 (2.6)	13.9 (1.1)	6.23 (1.34)
	FOS: 750 mg (75 mg/min)	5	161.0 (15.3)	0.17 (0.00)	0.30 (0.07)	50.4 (4.9)	50.7 (5.0)	13.3 (1.3)	5.77 (1.44)
Plasma total phenytoin	30-min infusion								
	PHT: 250 mg (8.3 mg/min)	10	7.60 (1.99)	0.45 (0.11)	16.0 (3.8)	118 (32)	127 (40)	1.94 (0.47)	42.9 (6.0)
	FOS: 375 mg (12.5 mg/min)	10	5.97 (0.70)	0.82 (0.17)	12.6 (2.9)	104 (27)	110 (32)	2.24 (0.48)	39.3 (3.6)
	FOS: 563 mg (18.75 mg/min)	6	9.12 (0.60)	0.72 (0.09)	14.5 (3.9)	199 (41)	208 (49)	1.75 (0.38)	35.2 (3.9)
	FOS: 750 mg (25 mg/min)	6	11.84 (1.23)	0.75 (0.09)	15.7 (3.9)	290 (59)	306 (71)	1.59 (0.36)	35.0 (6.1)
	10-min infusion								
	FOS: 563 mg (56.3 mg/min)	6	11.28 (1.91)	0.36 (0.07)	13.8 (3.0)	189 (18)	195 (21)	1.81 (0.20)	35.6 (5.8)
	FOS: 750 mg (75 mg/min)	5	13.66 (3.50)	0.33 (0.00)	16.5 (1.8)	283 (33)	298 (39)	1.58 (0.21)	37.2 (1.6)
Plasma free phenytoin	30-min infusion								
	PHT: 250 mg (8.3 mg/min)	10	0.55 (0.16)	0.43 (0.09)	17.7 (5.5)	6.9 (2.4)	8.7 (2.6)	28.3 (7.8)	686 (160)
	FOS: 375 mg (12.5 mg/min)	10	0.46 (0.08)	0.77 (0.16)	15.9 (3.8)	5.4 (1.7)	7.9 (2.3)	31.9 (9.7)	693 (96)
	FOS: 563 mg (18.75 mg/min)	6	0.70 (0.07)	0.58 (0.09)	15.9 (3.2)	11.0 (3.0)	12.6 (3.0)	29.1 (6.7)	645 (78)
	FOS: 750 mg (25 mg/min)	6	1.14 (0.16)	0.53 (0.07)	17.3 (3.5)	19.9 (3.8)	22.4 (4.3)	21.5 (4.8)	523 (70)
	10-min infusion								
	FOS: 563 mg (56.3 mg/min)	6	0.89 (0.19)	0.33 (0.00)	17.7 (3.8)	10.0 (2.1)	13.0 (1.4)	27.1 (3.0)	682 (115)
	FOS: 750 mg (75 mg/min)	5	1.33 (0.24)	0.30 (0.08)	16.4 (2.16)	19.8 (1.3)	21.9 (1.7)	21.4 (1.5)	508 (82)

*FOS* intravenous fosphenytoin sodium, *PHT* intravenous phenytoin sodium

Values are mean (standard deviation)

Plasma total fosphenytoin concentrations decreased by half in 0.24–0.31 h (14.4–18.6 min) after administration of fosphenytoin sodium, and to or below the lower limit of quantitation 2 h after the start of administration.

The mean *C*_max_/kg weight of plasma total phenytoin increased in a dose-dependent manner (α = 0.04, β = 1.03, *r* = 0.96 over a 30-min infusion). The mean AUC_*t*_/kg weight increased slightly more than proportional to the administered dose (α = −96.2, β = 34.0, *r* = 0.95 over a 30-min infusion), whereas the mean CL decreased with increasing dose. The mean *t*_1/2_ ranged from 12.6 to 16.5 h and increased slightly with increasing dose.

The *t*_max_ for plasma free phenytoin was generally shorter than that for plasma total phenytoin. However, nearly the same tendency was observed in the relationship between dose and each pharmacokinetic parameter for free and total phenytoin.

The max of the mean % unbound phenytoin is shown in Table 6. The profile (over time) of % unbound phenytoin for 4 h after administration was significantly different for fosphenytoin sodium (*P* = 0.0002). The profile of % unbound phenytoin for 750 mg fosphenytoin sodium (10-min infusion) transited higher when compared to other doses.

**Table 6:** Maximum of mean % unbound fraction of phenytoin

	Drug: dose (infusion rate)	*n*	Albumin (g/dL)	Max of mean % unbound phenytoin	Time after administration (min)^a^
Plasma phenytoin	*30-min infusion*				
	PHT: 250 mg (8.3 mg/min)	10	4.3 (0.28)	7.49 (0.95)	20
	FOS: 375 mg (12.5 mg/min)	10	4.3 (0.35)	8.65 (1.21)	30
	FOS: 563 mg (18.75 mg/min)	6	4.3 (0.40)	8.57 (0.44)	30
	FOS: 750 mg (25 mg/min)	6	4.3 (0.29)	11.30 (1.56)	30
	*10-min infusion*				
	FOS: 563 mg (56.3 mg/min)	6	4.3 (0.13)	10.31 (1.87)	10
	FOS: 750 mg (75 mg/min)	5	4.2 (0.19)	14.63 (1.90)	10

*FOS* intravenous fosphenytoin sodium, *PHT* intravenous phenytoin sodium

Values are mean (standard deviation)

^a^Time after administration to reach at maximum of mean % unbound phenytoin

**Fig. 3:**
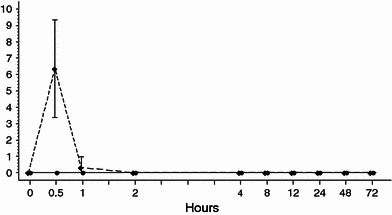
Time course of the infusion-site pain score (mean ± standard deviation) by the visual analog scale (VAS) after intravenous administration of fosphenytoin sodium *filled square* and phenytoin sodium *filled diamond* in healthy Japanese subjects in a randomized, double-blind, crossover study (study 1). At each time point, each subject marked a (slash) on a 10-cm scale, with the left side labeled “no pain” and the right side labeled “most severe pain,” at the point that corresponded to the level of pain intensity felt at that time

In study 1, all 12 subjects who received 250 mg phenytoin sodium experienced infusion-site pain, with a mean maximum VAS score of 6.4 ± 3.0, whereas such symptoms were not observed in subjects who received 375 mg fosphenytoin sodium (VAS 0 ± 0, Fig. 3). Of subjects who received 250 mg phenytoin sodium, 6 reported infusion-site burning and 3 subjects reported infusion-site itching. In subjects exhibiting infusion-site reactions, the mean maximum VAS score was 3.3 ± 2.9 for burning and 1.3 ± 1.0 for itching. No subjects reported these infusion-site symptoms during administration of 375 mg fosphenytoin sodium. The mean maximum score assigned to swelling, erythema, and tenderness by a physician was 2.0 (*n* = 1), 3.0 ± 1.4 (*n* = 4), and 2.0 (*n* = 1), respectively, in subjects who received 250 mg phenytoin sodium, whereas no subjects had such injection-site reactions after administration of 375 mg fosphenytoin sodium.

In study 2, in a total of 23 occasions in 13 subjects who received fosphenytoin sodium at doses of 563 or 750 mg or both, none exhibited infusion-site reactions, with the exception of one subject with infusion-site pain (VAS 0.1) and two individuals with infusion-site itching (VAS 0.4 and 0.5, respectively).

Significant adverse events (i.e., moderate or severe infusion-site pain requiring treatment discontinuation) were observed in 2 subjects who received 250 mg phenytoin sodium within 10 min after the start of administration in study 1. Superficial thrombophlebitis also developed in a subject who received 250 mg phenytoin sodium on the day after completion of treatment. In addition to these cases, a subject showed decreased blood pressure to 77/37 mmHg during infusion. Only one mild adverse event (common cold syndrome) was seen in 10 subjects administered 375 mg fosphenytoin sodium.

In study 2, one of the six subjects who received 563 mg fosphenytoin sodium (10-min infusion) experienced swelling with erythema and itching on the right palm, wrist, and instep (the same side as the infusion site) 4 h after administration. In addition, one subject who received 563 mg fosphenytoin sodium (10-min infusion) developed vertigo 13 min after initial administration. In this subject, nystagmus upon lateral gaze and staggered gait were observed 31 min after administration. None of the 11 subjects who received 750 mg fosphenytoin experienced moderate or severe adverse events.

**Fig. 4:**
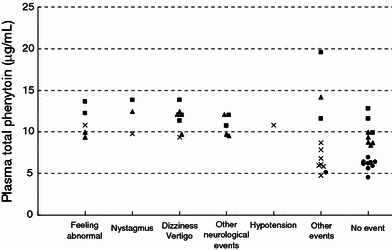
Correlation between adverse events and *C*_max_ (μg/mL) of plasma total phenytoin after intravenous administration of fosphenytoin sodium and phenytoin sodium for studies 1 and 2. *Cross marks* indicates phenytoin sodium 250 mg for study 1 (two events which were categorized a “other neurological event” and a “other event,” with missing concentration measurements due to discontinuation of administration were excluded in this figure). *Closed circles* indicate fosphenytoin sodium 375 mg for study 1. *Closed triangle* indicates fosphenytoin sodium 563 mg regardless of infusion rates for study 2. *Closed squares* indicates fosphenytoin sodium 750 mg regardless of infusion rates for study 2

The *C*_max_ of plasma total phenytoin concentrations was plotted against individual adverse events reported from study 1 and study 2 (Fig. 4). Neurological symptoms such as nystagmus and dizziness arose if the plasma total phenytoin concentration was ≥10 μg/mL.

ECG and vital signs were normal. No serious adverse events were reported in subjects receiving fosphenytoin sodium or phenytoin sodium.

## Discussion

The AUC of total and free phenytoin in subjects administered fosphenytoin sodium at a dose of 375 mg was very similar to those of phenytoin sodium administered at a dose of 250 mg (AUC_0–*t*_ ratio: 0.98 and 1.02, respectively), although the *C*_max_ of total and free phenytoin was lower for fosphenytoin sodium than for phenytoin sodium. In subjects, fosphenytoin is therefore almost completely converted to phenytoin. Browne et al. (1989) reported a similar AUC ratio of plasma total phenytoin of 0.992 in 12 subjects administered 375 mg fosphenytoin sodium and 250 mg phenytoin sodium in a crossover manner, which is consistent with the results of the present study.

When fosphenytoin sodium was administered at doses of 375, 563, and 750 mg, the conversion *t*_1/2_ of plasma total fosphenytoin was found to be 0.24–0.31 h, and thus was not considered to be related to dose or infusion rate. Eldon et al. (1993) reported a conversion *t*_1/2_ of 0.17–0.35 h, which was not influenced by dose and infusion rate when fosphenytoin sodium (at doses of 600, 1,200, and 1,800 mg) and placebo were infused at 18.8, 37.5, 75, 150, and 225 mg/min. These results are consistent with the results of the present study.

After administration of fosphenytoin sodium at doses of 375, 563, and 750 mg, the *C*_max_ of plasma total phenytoin increased in a dose-dependent manner, AUC increased more than proportionally with dose, and *t*_1/2_ also increased with increasing dose. The percent unbound fraction of phenytoin was higher at the highest dose (750 mg) of fosphenytoin sodium than at lower doses. This can be explained by the fact that more than 95 % of fosphenytoin sodium is tightly bound to plasma protein (Hussey 1990), and binds to albumin at the same site as phenytoin (Lai 1995), leading to displacement of phenytoin and resulting in a higher percent of unbound phenytoin (Eldon 1993). Phenytoin metabolism is known to be saturable (Gerber 1972; Jusko et al. 1976), which is believed to be the cause of the nonlinearity in the AUC and *t*_1/2_. The result of the percent unbound fraction is consistent with results from a study by Lai et al. (1995) using human plasma in vitro, which showed that the percent unbound fraction of phenytoin is related to plasma fosphenytoin concentration. However, the apparent CL decreased with increasing dose in the present study, whereas the percent unbound fraction increased when higher doses of fosphenytoin sodium were administered. For drugs such as phenytoin that possess a low metabolic capacity relative to the administered dose, if *Q* is much greater than (fu_b_ × CL_int_), then CL_organ_ ≅ fu_b_ × CL_int_ (Eq. 1), where *Q* is blood flow in the liver, fu_b_ is the unbound fraction of drug to protein in the blood (Benet 2010), and CL_int_ is the hepatic clearance of the drug. If apparent CL is approximately equal to CL_organ_ (Wilkinson and Shand 1975) and CL_int_ is constant because of the saturation of metabolism in the liver, an increase in fu_b_ should result in a proportional increase in apparent CL according to Eq. 1. However, this was not the case in the present study. The results of the present study indicate that CL_int_ did not remain constant, but decreased instead. In fact, a dose-dependent decrease in CL of plasma free phenytoin was observed. The possibility that an increase in the administered dose may cause a decrease in metabolic capacity is suggested. Birkett (1994) stated that the saturation of elimination mechanisms (which phenytoin exhibited) caused a change in CL_int_. This phenomenon appeared as a result of changes in AUC, *t*_1/2_, CL, and other parameters observed in the present study if the fosphenytoin sodium dose was increased from 375 to 563 mg. This finding suggests that metabolic capacity was influenced when intravenous fosphenytoin sodium was administered at a dose of at least 563 mg.

Fosphenytoin sodium had clearly superior tolerability at the infusion site compared with phenytoin sodium. All 12 subjects who received phenytoin sodium reported pain and other sensory symptoms at the infusion site, and 2 of these subjects discontinued treatment because of pain within 10 min after the start of administration. Furthermore, one subject experienced superficial thrombophlebitis. No subjects administered 375 mg fosphenytoin sodium experienced infusion-site adverse events. If pain intensity was rated using a 10-cm VAS scale (0 = none to 10 = severe pain), the maximum mean score was 6.4 ± 3.0 for subjects administered phenytoin sodium, whereas the score was 0 in subjects administered fosphenytoin sodium. Jamerson (1994) assessed pain using a 10-point ordinal scale (0 = none to 10 = severe) in 12 healthy American subjects. They reported that all subjects administered phenytoin sodium complained of pain, with a mean score of 5.83 ± 0.85 (standard error), whereas only two subjects administered fosphenytoin sodium had a score of 1 and 2. These results are essentially consistent with those of the present study.

In a total of 33 occasions in 23 subjects administered fosphenytoin sodium at doses of 375, 563, and 750 mg, the most frequently reported adverse events were dizziness in 4 subjects and abnormal feeling in 4 subjects. However, most of these symptoms were mild in severity and transient. Moderate adverse events were observed in 2 subjects administered fosphenytoin sodium 563 mg (10-min infusion), which included vertigo and swelling in the extremities. Kutt et al. (1964) reported that the onset of nystagmus occurred at ≥20 μg/mL, the onset of ataxia at ≥30 μg/mL, and the onset of somnolence or psychiatric symptoms at ≥40 μg/mL. In the present study, nystagmus was observed in 2 subjects, but their peak plasma total phenytoin concentrations (12.4 and 13.8 μg/mL, respectively) were much lower than the toxic concentration.

In conclusion, fosphenytoin sodium was rapidly converted to phenytoin after single intravenous administration of 375 mg fosphenytoin sodium in healthy Japanese male subjects. Fosphenytoin sodium showed lower *C*_max_ values for plasma total phenytoin, but equivalent AUC, compared with phenytoin sodium. In addition, after a single intravenous dose of fosphenytoin sodium at doses of 375, 563, and 750 mg, the *C*_max_ of plasma total phenytoin increased proportionally with increasing dose, but AUC, *t*_1/2_, and CL showed nonlinearity. In contrast to phenytoin, almost no adverse events at the infusion site were observed after administration of fosphenytoin sodium. Fosphenytoin sodium was well tolerated at doses up to 750 mg and at infusion rates up to 75 mg/min. Further studies on the effects of higher doses and increased infusion rates are required.
